# Stemness-Attenuating miR-503-3p as a Paracrine Factor to Regulate Growth of Cancer Stem Cells

**DOI:** 10.1155/2018/4851949

**Published:** 2018-04-04

**Authors:** Minkoo Seo, Seung Min Kim, Eun Young Woo, Ki-Cheol Han, Eun Joo Park, Seongyeol Ko, Eun wook Choi, Mihue Jang

**Affiliations:** ^1^Prostemics Research Institute, Seongdong-gu, Seoul 135-010, Republic of Korea; ^2^Center for Theragnosis, Biomedical Research Institute, Korea Institute of Science and Technology, Seongbuk-Gu, Seoul 136-791, Republic of Korea

## Abstract

Cancer stem cells (CSCs) with self-renewal abilities endorse cellular heterogeneity, resulting in metastasis and recurrence. However, there are no promising therapeutics directed against CSCs. Herein, we found that miR-503-3p inhibited tumor growth via the regulation of CSC proliferation and self-renewal. miR-503-3p, isolated from human adipose stem cell- (ASC-) derived exosomes, suppressed initiation and progression of CSCs as determined by anchorage-dependent (colony formation) and anchorage-independent (tumorsphere formation) assays. The expression of pluripotency genes was significantly decreased in miR-503-3p-treated CSCs. Furthermore, xenografts, which received miR-503-3p, exhibited remarkably reduced tumor growth *in vivo*. Thus, miR-503-3p may function as a stemness-attenuating factor via cell-to-cell communications.

## 1. Introduction

Exosomes are secretory vesicles that are composed of lipid bilayer membranes, ranging from 30 to 100 nm in diameter [1–3]. Exosomes are naturally produced in numerous cells and released into the extracellular membrane. In the past, exosomes were considered to function in the cellular release of cell debris and toxins [4]. However, recently, there has been great interest in exosomes with respect to clinical therapeutics and drug delivery [5–7]. Exosomes participate in multiple biological processes, including signal transduction, tumorigenesis, immune responses, and apoptotic processes [8–10]. In addition, exosomes may serve as potential biomarkers for cancer diagnosis and prognosis and as a therapeutic target for cancer treatment. Exosomes can deliver a variety of molecules, including growth factors, proteins, DNA, mRNA, miRNA, and noncoding RNA, which can act as autocrine and paracrine factors [11, 12]. Thus, exosomes can act as crucial communicators in diverse biological processes.

Stem cells are capable of self-renewal and differentiating into a variety of cell types. Mesenchymal stem cells (MSCs) can affect microenvironments by secreting exosomes in an autocrine and paracrine manner [13]. MSC-derived exosomes play distinct roles in tumor signaling, growth, and metastasis [14–17]. However, the effects of exosomes, contained in stem cells, are still controversial. The different effects of exosomes depend on their tissue of origin and cancer cell type [18]. For instance, human MSC-derived exosomes play a critical role in cell proliferation and metastasis of breast cancer, suggesting existence of numerous tumor-supportive miRNAs and factors, such as platelet-derived growth factor receptor-*β* and tissue inhibitor of metalloproteinase- (TIMP-) 1 and TIMP-2 in hMSC-derived exosomes [14]. MSC-derived exosomes also promote tumor growth and progression by activating extracellular signal-regulated kinase 1/2 (ERK 1/2) [15]. In addition, exosomes from the bone marrow and umbilical cord stem cells suppress cell proliferation [18], whereas stem cell-derived exosomes can exert anticancer effects [19–22]. Interestingly, intratumoral injection of exosomes, derived from miR-146b expressing MSCs, induced a significant reduction in cancer growth in the rat primary brain tumor model [20]. This suggests that miRNAs, possessing anticancer activity, can be packaged into exosomes for potential clinical applications.

Recent studies indicate that miRNAs can regulate population and progress of cancer stem cell (CSCs). miRNAs are short noncoding RNAs that degrade or attenuate the translation of target mRNA by imperfectly binding to their 3′-untranslated region (UTR), thereby playing considerable roles in human cancers [23, 24]. miRNAs expressed in all animal cell types may regulate the potential for self-renewal and differentiation in normal stem cells. Stemness-attenuating miRNAs can regulate tumor initiation and development. Recently, miR-203, which targets *survivin* and *Bmi-1*, was shown to inhibit proliferation and self-renewal of leukemia stem cells [25], and miR-34a suppresses the development of malignant glioma stem cells [26]. miR-199-5p also inhibits tumor growth by blocking Notch signaling, which inhibits the self-renewal ability of the CD133^+^ population [27]. In contrast, aberrant expression of certain miRNAs in CSCs promotes cancer initiation and development. miR-21 is highly accumulated in CSCs, including glioblastoma and colon CSCs, and plays key roles in apoptosis and proliferation of CSCs [28]. miR-21, in the cancer progenitor cell niche, can promote cancer stem/progenitor cell populations by directly regulating progenitor cells to self-renew and by triggering production of growth factors in nonprogenitor cancer cells, thereby enriching stem cell populations [29, 30]. Dysregulated miRNAs can be important signatures involved in the activation of self-renewal, tumor progress, and resistance to anticancer therapies. Thus, manipulation of tumor-supportive or tumor-suppressive miRNA, which regulates the properties of self-renewal and differentiation, gives rise to great potential for cancer therapeutics against CSCs.

Herein, to investigate the roles of stem cell-derived exosomes in tumorigenesis, we analyzed a variety of enriched miRNAs, which may function as tumor-supportive or suppressive factors, in exosomes isolated from ASCs. Among the RNA cargoes identified by a miRNA microarray analysis, we found that miR-503-3P inhibited stemness via downregulation of Nanog, a pluripotent marker, and upregulation of *CK-18*, a differentiation marker, resulting in significantly reduced tumor growth *in vivo*.

## 2. Materials and Methods

### 2.1. Cell Cultures

The human breast cancer cell lines MCF7, BT-474, HCT-15, and COLO 205 were obtained from the Cell Bank of American Type Culture Collection (ATCC, USA). Cells were routinely cultured in RPMI-1640 medium supplemented with 10% fetal bovine serum (FBS) (Thermo Scientific, USA), 100 U/mL penicillin, and 100 *μ*g/ml streptomycin at 37°C in a humidified atmosphere containing 5% CO_2_.

For adipose stem cell (ASC) culture, samples of human subcutaneous adipose tissue were obtained from elective liposuction of healthy females, with informed consent, as approved by the institutional review boards. Primary ASCs were derived from a 20-year-old female, and all related experiments were performed using ASCs at passage 4. The tissues were digested with 0.075% collagenase type II (Sigma-Aldrich, USA) under gentle agitation for 45 minutes at 37°C and centrifuged at 300 ×g for 10 minutes to obtain the stromal cell fraction. The pellet was filtered through a 70 *μ*m nylon mesh filter and resuspended in phosphate buffered saline (PBS). The cell suspension was layered onto histopaque-1077 (Sigma-Aldrich, USA) and centrifuged at 840 ×g for 10 minutes. The cell band, floating over the Histopaque, was collected; the retrieved cell fraction was cultured at 37°C in DMEM supplemented with 10% FBS, 100 U/mL of penicillin, and 100 *μ*g/mL streptomycin. ASCs were used for experiments at passage 4.

### 2.2. Purification of Exosomes

Human ASCs were isolated and cultured as previously described [31]. Upon reaching 80% confluence, the cells were washed three times with PBS and resupplemented with serum-free DMEM/F12 (Welgene, Korea) for 48 h. Then ASC-conditioned media were harvested for further experiments. To remove the cells and debris, the harvested media was sequentially centrifuged at 300*g* and 3000*g*, respectively, at 4°C for 10 min using a high-speed centrifuge 1736R (LaboGene, Seoul, Korea). Supernatant was ultracentrifuged at 110,000*g* for 70 min using Optima XE-90 Ultracentrifuge (Beckman Coulter, Brea, CA, USA), and then the pellets were carefully resuspended in PBS.

### 2.3. Characterization of ASC-Derived Exosomes

To detect exosomal morphology using transmission electron microscopy, 100 *μ*L of exosome suspension in 1x PBS was air-dried onto 200-mesh Formvar-carbon-coated copper grids (Ted Pella, Redding, CA), negatively stained with 2% aqueous uranyl acetate, and observed with 100 kV transmission electron microscope (TEM) (HITACHIH-7650, Japan).

To measure the distribution of exosomal size and concentration, we used a dynamic scattering laser (NanoSight NS300, Malvern, UK) and Nanoparticle Tracking Analysis software version 2.2 (NanoSight). We then diluted 1 mg/mL of exosomes in 1 mL PBS, and each sample was measured three times independently.

### 2.4. Microarray Analysis

The conditioned ASC media, as described in Section 2.2 *. Purification of exosomes* was prepared for microarray analysis. To examine the microRNA expression profile in ASC-derived exosomes, conditioned ASC media were harvested at 48 h postincubation, and exosomes were purified for microarray analysis. For negative control, conditioned media, from ASCs without exosomes (w/o exosome), were used by removing the exosomes after centrifugation at 110,000 ×g for 2 h. Total RNAs were extracted from the ASC-derived exosomes using the Total Exosome RNA and Protein Isolation Kit (Invitrogen, USA) according to the manufacturer's protocol. The quantity of the RNA was measured using an Agilent BioAnalyzer™ 2100 (Agilent, USA). A microRNA microarray was performed by a commercial service company (Biocore Inc., Korea) using an Affymetrix GeneChip® miRNA 4.0 array (*Homo sapiens*). The sample labeling, microarray hybridization, and washing were conducted in accordance with the manufacturer's standard protocols. Briefly, the total exosome RNA was labeled using the FlashTag™ Biotin HSR RNA Labeling Kit. Then the labeled RNAs were hybridized onto the microarray. After washing and staining the slides, the microarrays were scanned using the GeneChip Scanner 3000 G7. An Affymetrix® GeneChip Command Console® software (AGCC) was used for the following data analysis.

### 2.5. Isolation of CD44^+^ CSCs

To isolate CD44-positive CSCs, 1 × 10^7^ cancer cells were collected during the exponential growth phase and centrifuged at 1000 rpm for 5 min. After centrifugation, pellets were suspended with 160 *μ*L of DPBS and labeled using 40 *μ*L of monoclonal anti-CD44 antibody coupled with magnetic beads (Miltenyi Biotec, Germany) in cold MACS running buffer. Cells were incubated for 15 min at 4°C and then washed with DPBS several times. Cells, resuspended in DPBS, were passed through an LS column (Miltenyi Biotec, Germany) and rinsed three times with 5 mL of MACS buffer. The column was removed from the separator and placed on a suitable collection tube. The magnetically labeled CD44^+^ cells were flushed out using the plunger supplied with the column. For the culture of CD44^+^ CSCs, the cells were maintained in DMEM/F12 (Welgene, Korea) containing 10% N21 supplement (R&D system, USA), 1% penicillin streptomycin (Welgene, Korea), 20 ng/mL human bFGF-2 (Thermo Scientific, USA), and 20 ng/mL EGF (Stemcell Technologies, CA).

### 2.6. Colony Formation Assay

Cells were seeded into 6-well plates, at a density of 2 × 10^3^ cells per well, in complete media. After 24 h, 20 nM of scrambled miRNA (miR-NC), miR-328-3p, and miR-503-3p was transfected as described above, and the cells were grown up to 3 weeks by replacing the media with fresh media every 3 days. Then colonies were stained with a solution including 0.5% crystal violet and 25% methanol for 30 min and washed with tap water three times. The colonies were counted using ImageJ software. miRNA sequences, used for transfection, are as follows:

miRNA-NC sense: 5′- CCUCGUGCCGUUCCAUCAGGUAGUU -3′

miRNA-NC antisense: 5′-CUACCUGAUGGAACGGCACGAGGUU-3′

miR-328-3p sense: 5′-CUGGCCCUCUCUGCCCUUCCGU-3′

miR-328-3p antisense: 5′-ACGGAAGGGCAGAGAGGGCCAG-3′

miR-503-3p sense: 5′-GGGGUAUUGUUUCCGCUGCCAGG-3′

miR-503-3p antisense: 5′-CCUGGCAGCGGAAACAAUACCCC-3′

### 2.7. qRT-PCR

Cells, transfected as described above, were harvested by Accutase (Thermo Scientific, USA), and total RNA was purified with an RNeasy Mini kit (QIAGEN, GmBH, Germany) following the manufacturer's instructions. To validate the expression of mRNAs by quantitative real-time PCR (qRT-PCR), 1 *μ*g of total RNA was reverse-transcribed using One Step PrimeScript RT-PCR kit (Takara, Japan). qRT-PCR was conducted using a StepOnePlus Real-Time PCR System (Applied Biosystems, US). The primer sequences, used for qRT-PCR, are as follows:

Nanog forward: 5′-CCCCAGCCTTTACTCTTCCTA-3′

Nanog reverse: 5′-CCAGGTTGAATTGTTCCAGGTC-3′

OCT4 forward: 5′-GTGTTCAGCCAAAAGACCATCT-3′

OCT4 reverse: 5′-GGCCTGCATGAGGGTTTCT-3′

CK-18 forward: 5′-TGAGACGTACAGTCCAGTCCTT-3′

CK-18 reverse: 5′-GCTCCATCTGTAGGGCGTAG-3′

### 2.8. MTT Assay

MCF7 cells were seeded into a 24-well plate. The cells were grown to 50–70% confluence and then treated with ASC exosomes or transfected with 20 nM miRNA, using Lipofectamine™ 2000 (Invitrogen, Carlsbad, CA, USA) for 3 days, according to the manufacturer's protocol. This step was repeated five times. Cell viability was determined by an MTT assay.

### 2.9. Spheroid Body Formation Assay

When primary tumor spheres reached the population of approximately 200–500 cells per spheroid body, the spheroid bodies were dissociated at a density of 1000 cells per mL; then each suspension of 100 single cells was seeded into a 24-well plate in serum-free media. Cells were transfected, with 20 nM miR-NC or miR-503-3p, using Lipofectamine 2000 (Invitrogen, USA) according to the manufacturer's instructions. After 1 week, the cells were analyzed for spheroid body formation and quantified using an inverted microscope (EVOS). miRNA sequences, used for transfection, are as follows:

miR-503-3p mutant sense: 5′-GCCCAUAAGUUUCCGCUGCCAGG -3′

miR-503-3p mutant antisense: 5′-CCUGGCAGCGGAAACUUAUGGGC-3′

### 2.10. Luciferase Reporter Assay

The 3′-untranslated region (UTR) fragment of Nanog (NM_024865.3) was amplified from the genomic DNA of MCF7 cells and cloned between the Renilla luciferase coding sequence and the poly(A) site of the psiCHECK-2 plasmid (Promega, Madison, WI, USA), using XhoI/NotI sites to produce psiC-Nanog. The primers, used for the amplification of 3′-UTR of Nanog, were as follows: 5′-CTCGAGctccatgaacatgcaacctg-3′ and 5′-GCGGCCGCcactcggtgaaatcagggtaa-3′. A luciferase reporter assay was conducted to test whether miR-503-3p directly targeted the 3′-UTR of Nanog. Briefly, MCF7 cells were seeded into a 96-well plate (5 × 10^3^ cells/well). After 24 h, cells were cotransfected with 20 ng psiC-Nanog and 10 nM of the miRNA mimics. Luciferase activity was measured 48 h posttransfection using the Dual-Glo™ luciferase reporter assay system (Promega). Luciferase activity was normalized to the activity of internal firefly luciferase, for each sample, and quantified with respect to the ratio of luciferase activity obtained from the scrambled miRNA-transfected cells.

### 2.11. In Vivo Experiments

All animal maintenance and *in vivo* experiments were conducted according to the regulations of the Institutional Animal Care and Use Committee of the Korea Institute of Science and Technology and KNOTUS IACUC (approval number 2016-057 and number #KNOTUS IACUC 16-KE-154). For the generation of MCF7 xenografts, 1 × 10^7^ cells were suspended in a 50% Matrigel solution (BD Biosciences) and injected subcutaneously into nude mice. When the tumors reached ~0.1 cm^3^, 2 mg/mL of miR-503-3p and miRNA-NC was administered intratumorally into the xenografts six times every 3 days. Tumor volumes were monitored every 3 days for 4 weeks and determined by the formula V = (length × width^2^). The mice were sacrificed at 28 days posttreatment. For histological observation, tumor sections were stained with hematoxylin and eosin (H&E) according to the standard protocol and observed under a light microscope (Olympus). To detect apoptotic cells *in vivo*, each tumor section was fixed in 4% paraformaldehyde and permeabilized in 0.1% Triton X-100 and 0.1% sodium citrate. Then fixed tumor sections were accessed by transferase-mediated dUTP nick end labeling (TUNEL) assay using an in situ cell death detection kit (Roche) according to the manufacturer's instructions, and nuclei were visualized using DAPI mounting medium (Vector Laboratories). The apoptotic cells were monitored by a confocal microscopy and quantified using ImageJ software (NIH).

## 3. Results

### 3.1. ASC-Derived Exosomes Deliver a Variety of miRNAs

**Figure 1:**
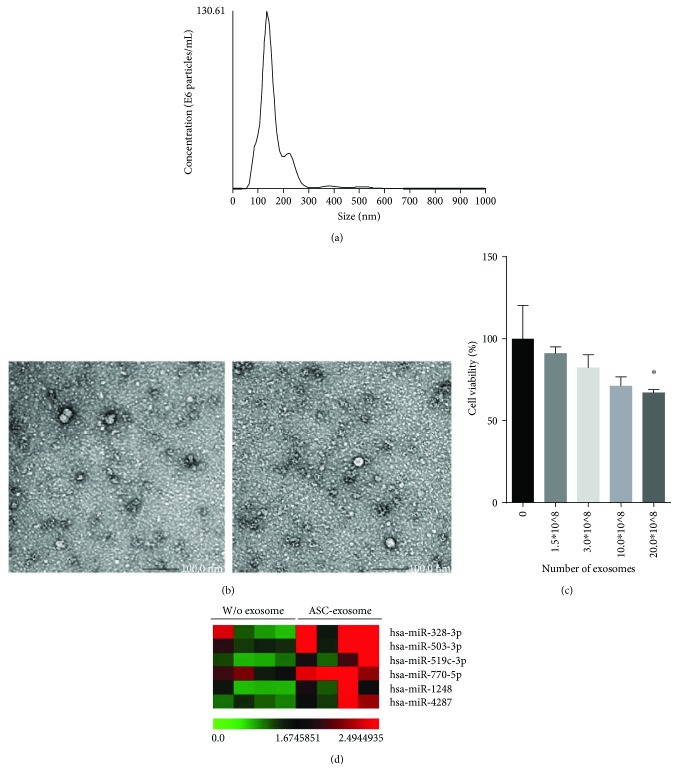
Characterization of human ASC-derived exosomes and their RNA cargo. The size and morphology of isolated membrane-bound exosomes are shown using (a) NTA analysis and (b) TEM images. ASC-derived exosomes show a size distribution ranging from 90 to 200 nm. The bars indicate 100 nm. (c) ASC-derived exosomes exert a cytotoxic effect, as determined by an MTT assay. ^∗^ *P* < 0.05 as analyzed by one-way ANOVA followed by Tukey's test. (d) A list of identified miRNA cargoes resident in ASC-derived exosomes is analyzed by a miRNA microarray. Color bar indicates fold change in gene expression; w/o exosomes and with ASC exosomes indicate conditioned media from ASCs without or with exosomes.

To investigate the role of ASC-derived exosomes in tumori genesis, we evaluated the anticancer effects of ASC-derived exosomes in MCF7 breast cancer cells (Figures 1(a)–1(c)). First, we isolated exosomes from human ASCs using ultracentrifugation method (Figures 1(a) and 1(b)). NTA and TEM analysis revealed that membrane-bound exosomes at a concentration of approximately 9.76 × 10^12^ were purified from 100 L of culture media. To assess anticancer efficacy of the ASC-derived exosomes, we tested cell viability after treating the cells with isolated ASC-derived exosomes (Figure 1(c)). ASC-derived exosomes (at 20.0 × 10^8^) showed anticancer activity with a 30% reduction in cell proliferation. To determine the RNA cargo contained in the exosomes and how this cargo may inhibit cancer growth, we conducted a microarray using an Affymetrix GeneChip miRNA 4.0 Array. The top six miRNAs (>1.5-fold; *P* < 0.05) are listed in Figure 1(d).

### 3.2. miR-503-3p Inhibits Colony-Forming Activity in Cancer

**Figure 2:**
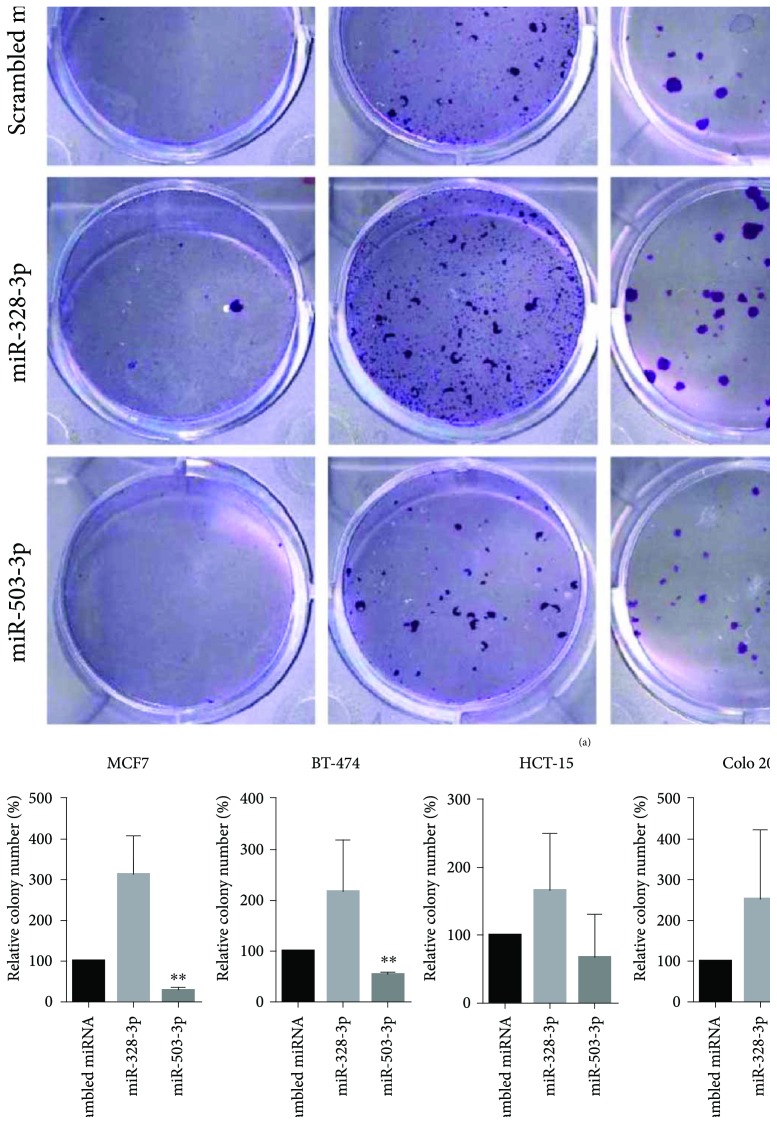
The effect of exosomal miRNA on colony formation in vitro. To evaluate the function of miR-328-3p and miR-503-3p on cancer stem-like properties, a colony formation assay was performed using four different cancer cell lines including MCF7, BT-474, HCT-15, and COLO 205. (a) The numbers of colonies are shown using cells transfected with 20 nM miR-328-3p and miR-503-3p. Treatment with miRNA-NC was used as negative control. Inserts indicate magnified images. (b) Colony numbers were quantified using ImageJ. The number of colonies, formed by cells transfected with miR-503-3p, was significantly lower than that formed by cells treated with negative control. ^∗∗^ *P* < 0.01 by a one-way ANOVA followed by Tukey's test.

Among the miRNAs, identified within the exosomes, miR-503-3p can regulate cell proliferation and apoptosis *via* direct targeting to p21, resulting in inhibition of cancer growth. Additionally, the cell-cycle inhibitor P21 is crucial for the self-renewal of leukemia stem cells [32]. Whole exosomes, isolated from ASCs, inhibited the cell viability of cancer cells (Figure 1(c)); therefore, we investigated the function of miRNAs contained in these exosomes. We used a colony formation assay to examine whether both miR-328-3p and miR-503-3p can suppress cancer stem cell- (CSC-) like phenotypes (Figure 2). When the four different cancer cell lines, including MCF7, BT-474, HCT-15, and COLO 205, were treated with miR-503-3p, the number of colonies was greatly reduced (Figure 2(b)). MCF7 cells had the lowest survival fraction at 28.3%. However, treatment with miR-328-3p increased survival fractions in the four different cancer cell lines. Stem cell-derived exosomes appear to both promote and inhibit tumor growth, depending on the conditions, tumor type, the origin of stem cells, the stage of tumor development, and the diverse tumor environment [33]. miRNAs play pivotal roles as key regulators in tumorigenic and tumor-suppressive processes, which may directly regulate certain oncogenes and tumor-suppressive genes [34]. miRNAs also contribute to the control of tumor-modifying extrinsic factors, such as the immune system, stromal cell interactions, and oncoviruses. Hence, the balance between oncogenic and tumor-suppressive processes may be influenced by overall behaviors of various miRNAs regulating the expression of oncogenes and tumor suppressors. Our miRNA microarray analysis (Figure 1(d)) indicated that ASC-derived exosomes contain a variety of miRNAs, which may act in an oncogenic or tumor-suppressive manner. Our colony formation assay (Figure 2) indicated that miR-503-3p and miR-328-3p had opposing effects on colony formation; the final biological effect of ASC-derived exosomes was inhibition of cancer growth (Figure 1(c)). The cytotoxic effects, mediated by ASC-derived exosomes, may be influenced by the inhibitory roles of miR-503-3p. Tumorigenesis was inhibited at significant expression of miR-503-3p (*P* < 0.01); however, other tumor-suppressive miRNAs were also expressed. These results indicate that miR-503-3p may be a key factor within ASC-derived exosomes and may show anticancer activity by controlling stem cell-like properties.

### 3.3. miR-503-3p Regulates Cancer Stemness

**Figure 3:**
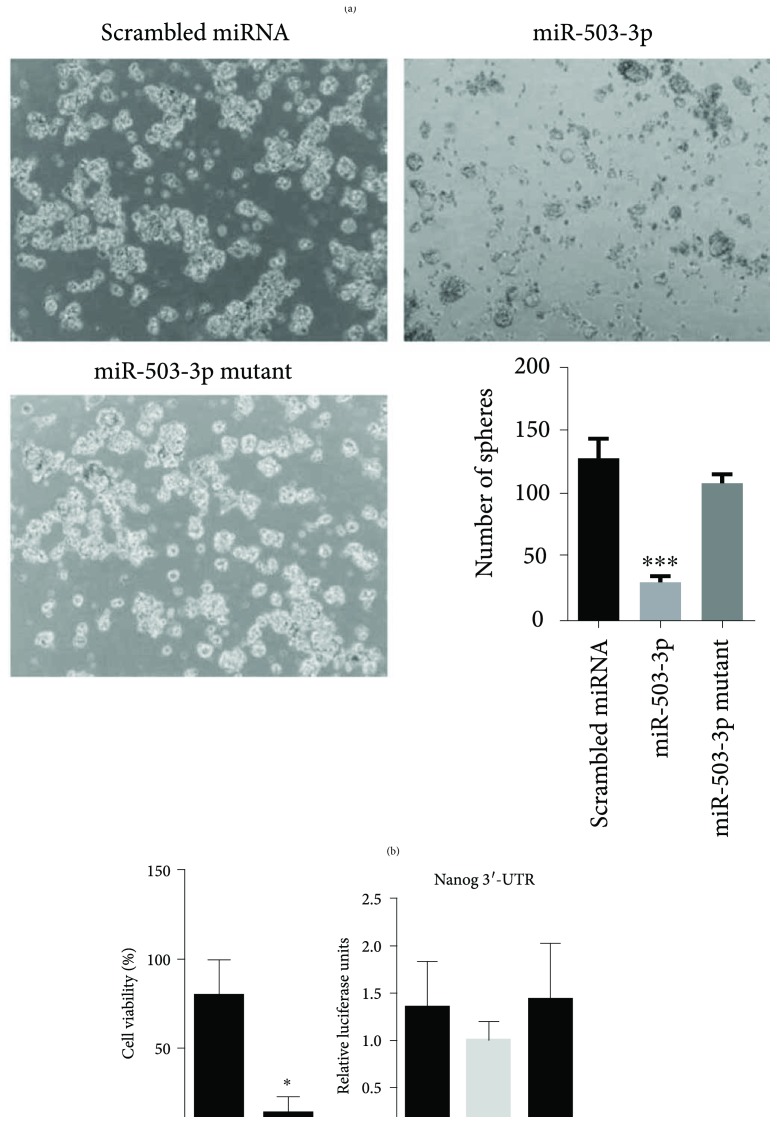
miR-503-3p inhibits stem cell-like properties in MCF7 cells. (a) qRT-PCR analysis showed that miR-503-3p significantly decreases the level of Nanog, a pluripotent transcription factor, in MCF7 CSCs. ^∗∗^ *P* < 0.01. (b) Tumor-sphere formation in MCF7 CSCs, transfected with miR-503-3p, is dramatically inhibited, compared to that of the negative control or cells treated with mutant miR-503-3p. Representative spheroids are shown in the left panels, and spheroid formation was quantified using ImageJ. ^∗^ *P* < 0.05 and ^∗∗∗^ *P* < 0.001 are determined by one-way ANOVA followed by Tukey's test. (c) Cytotoxic effects are analyzed by an MTT assay. miR-503-3p significantly reduces cell viability in MCF7 cells. (d) miR-503-3p indirectly regulates the expression of Nanog. Luciferase activity was measured in MCF7 cells cotransfected with psiC-Nanog and miR-503-3p mimics. Error bars indicate SDs (*n* = 3 per experiment). ^∗^ *P* < 0.05.

Next, we investigated the possibility of whether miR-503-3p plays inhibitory roles in self-renewal and survival of CSCs. For this, we used qRT-PCR to measure the expression of self-renewal and differentiation marker in MCF7 CSCs isolated from an estrogen receptor- (ER-) and progesterone- (PR-) positive MCF7 cells [35] (Figure 3). CD44^+^ CSCs from MCF7 were isolated using a monoclonal anti-CD44 antibody coupled with magnetic beads and confirmed by expression of endogenous Nanog (a CSC marker) after isolation of CSCs [36, 37] (Supplementary Figure 1). Next, the expression of Nanog, a pluripotency transcription factor, was evaluated in miRNA-negative control (miRNA-NC) or miR-503-3p-treated CSCs for 48 h and analyzed by qRT-PCR (Figure 3(a)). miR-503-3p significantly inhibited the level of Nanog at 30.5%. Conversely, cytokeratin 18 (CK18) mRNA, used as a differential stem cell marker, was highly increased in miR-503-3p-transfected NC-treated CSCs [38]. Treatment with miR-503-3p inhibited 69.7% of spheroid formation, which is a characteristic property of CSCs; spheroid formation was significantly lower than that of CSCs treated with mutant miR-503-3p (Figure 3(b)). The number of spheroids, formed by CSCs treated with mutant miR-503-3p, did not differ from that formed by the controls. In addition, 86.8% of cell viability was inhibited in miR-503-3p-treated CSCs (Figure 3(c)).

We used a luciferase reporter assay to test whether Nanog is a direct target of miR-503-3p (Figure 3(d)). The 3′-UTR fragment of Nanog was incorporated into the dual luciferase reporter vector and transfected, along with each miRNA mimic, into MCF7 cells, to validate the target gene. However, the activity of luciferase was not reduced even in the miR-503-3p-treated cells. Although the expression of Nanog can be regulated by miR-503-3p, our results indicate that Nanog is not a direct target of miR-503-3p, assuming that miR-503-3p indirectly regulates the expression of Nanog in its downstream pathways. Thus, further studies are needed to identify the direct target gene.

### 3.4. miR-503-3p Functions as an Anticancer Factor In Vivo

**Figure 4:**
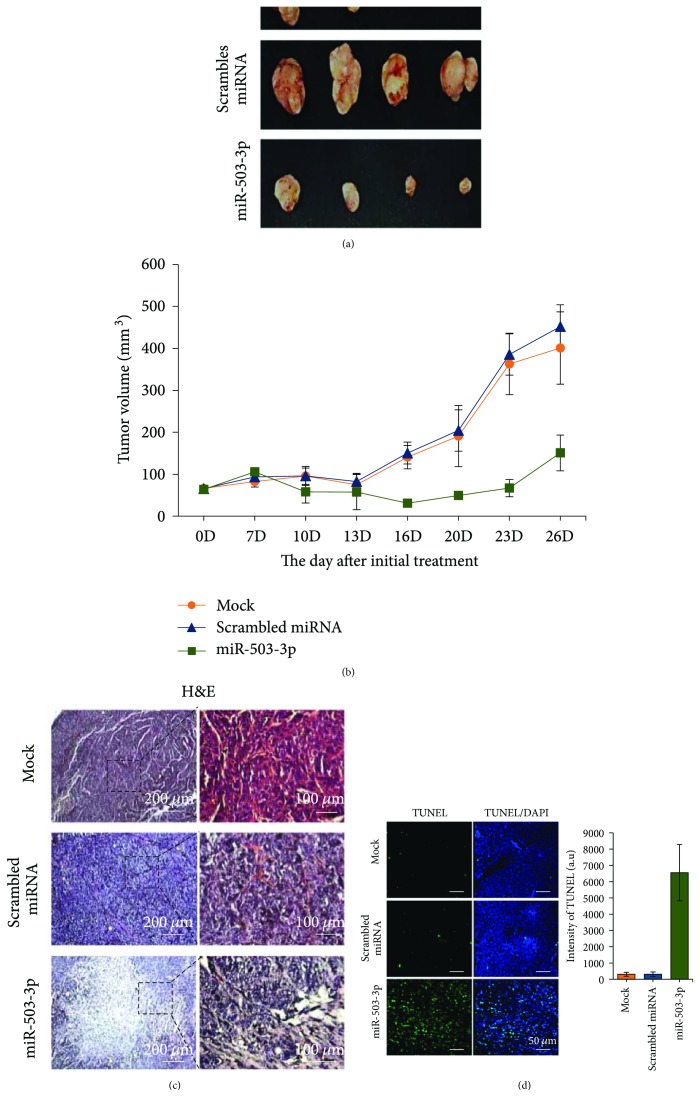
miR-503-3p exerts antitumor activity on MCF7 xenografts. (a) The dissected tumors are shown at 28 days postinitial treatment. (b) Tumor growth curves after initial treatment. miRNA-NC and miR-503-3p were intratumorally administered every 3 days for 2 weeks, and tumor volumes were monitored every 3 days. Error bars indicate SDs (*n* = 4). (c) Histologic observation was conducted using hematoxylin and eosin (H&E) staining. Right panels indicate the magnified images from the dotted-line boxes. (d) TUNEL assay was used to detect apoptotic cells in tissues. Apoptotic cells were visualized by confocal microscopy and quantified using ImageJ.

Based on the results of our *in vitro* functional assay, we speculated that miR-503-3p is involved in the regulation of CSC stemness. Thus, we investigated whether miR-503-3p can inhibit tumor growth in ER/PR-positive MCF7-bearing xenografts (Figure 4). The control (mock and miR-NC) treatment and miR-503-3p were intratumorally administered into MCF7 xenografts (Figure 4(a)). After initial treatment, the xenografts were monitored, and tumor volumes were measured using a digital caliper. At day 28, the dissected tumors, from the xenografts that received miR-503-3p, showed dramatic reduction (by 66%) in tumor growth (Figures 4(a) and 4(b)). Interestingly, large necrotic regions were observed in the tumors administered miR-503-3p compared with those in the tumors administered mock or miR-NC (Figure 4(c)). To detect the induction of apoptotic cells in tissues, we performed a TUNEL assay (Figure 4(d)). Expectedly, the administration of miR-503-3p triggered apoptosis, suggesting that miR-503-3p has antitumor activity.

## 4. Discussion

CSCs were first discovered in acute myeloid leukemia, and the existence of CSCs has been reported in numerous human cancer cell lines and tissues [39–42]. CSCs may play crucial roles in tumor initiation, progress, maintenance, and metastasis [43, 44]. CSCs show stem-like properties, with multipotent abilities such as self-renewal and the expression of pluripotent stem cell surface and transcription factor genes. Moreover, CSCs can differentiate into maturing cells with cellular heterogeneity, which show resistance to conventional therapy, can cause distant metastasis, and result in relapse [45]. CSCs do not respond to standard anticancer therapies, resulting in intrinsic or acquired resistance to anticancer drugs. Despite their potential clinical significance, their molecular and cellular mechanisms are not completely understood. Thus, it is crucial to elucidate the characteristics of CSCs in order to develop anticancer therapeutics targeting CSCs.

miR-503-3p was discovered in exosomes isolated from ASCs, suggesting that miR-503-3p may function as a paracrine factor in cell-to-cell communications. miR-503-3p appears to regulate CSC-like phenotypes by controlling pluripotent transcription factors. As a paracrine factor, miR-503-3p may be a key factor in the initiation and progression of CSCs. We investigated whether miR-503-3p is a direct target of Nanog. Although we used a bioinformatics analysis to determine the direct target molecules, we did not find promising data. However, aberrant expression of miR503-3p has been observed in various tumors and upregulation of miR-503-3p inhibits cancer viability, especially in 786-0 renal cancer; this indicates that miR-503-3p may function as an antioncogenic factor [46]. Moreover, RNA-Seq data demonstrated that some genes, such as p21 and CDK4, which are involved in CSC signaling pathways, are downregulated after treatment with miR-503-3p [46]. Thus, miR-503-3p may play an important role as a paracrine factor in the regulation of CSCs.

## 5. Conclusions

We found that miR-503-3p was highly enriched in exosomes derived from human ASCs, which inhibited the initiation and progression of CD44^+^ CSCs. The level of the pluripotency genes was significantly downregulated in miR-503-3p-transfected CSCs. Furthermore, xenografts, which received miR-503-3p, exhibited remarkably reduced tumor growth *in vivo*. Thus, miR-503-3p may function as a stemness-attenuating factor via cell-to-cell communications.

## Supplementary Materials

Supplementary Materials Supplementary Figure 1: characterization of isolated CD44^+^ CSCs. (A) MCF7 monolayer cells and isolated MCF7 CSCs using CD44-positive magnetic beads are shown. (B) Confocal microscopic images revealed the expression of Nanog as a stemness marker. The bars indicate 400 *μ*m. The nucleus was visualized by DAPI staining.
