# Supplementary material for: Stemness-Attenuating miR-503-3p as a Paracrine Factor to Regulate Growth of Cancer Stem Cells
Source: Stem Cells Int. 2018 Apr 4;2018:4851949. doi: 10.1155/2018/4851949 (PMC5904772; doi:10.1155/2018/4851949)
Supplement: Supplementary Materials — Supplementary Figure 1: characterization of isolated CD44+ CSCs. (A) MCF7 monolayer cells and isolated MCF7 CSCs using CD44-positive magnetic beads are shown. (B) Confocal microscopic images revealed the expression of Nanog as a stemness marker. The bars indicate 400 μm. The nucleus was visualized by DAPI staining. [file 4851949.f1.pdf]

Supplementary Materials

Supplementary Figure 1

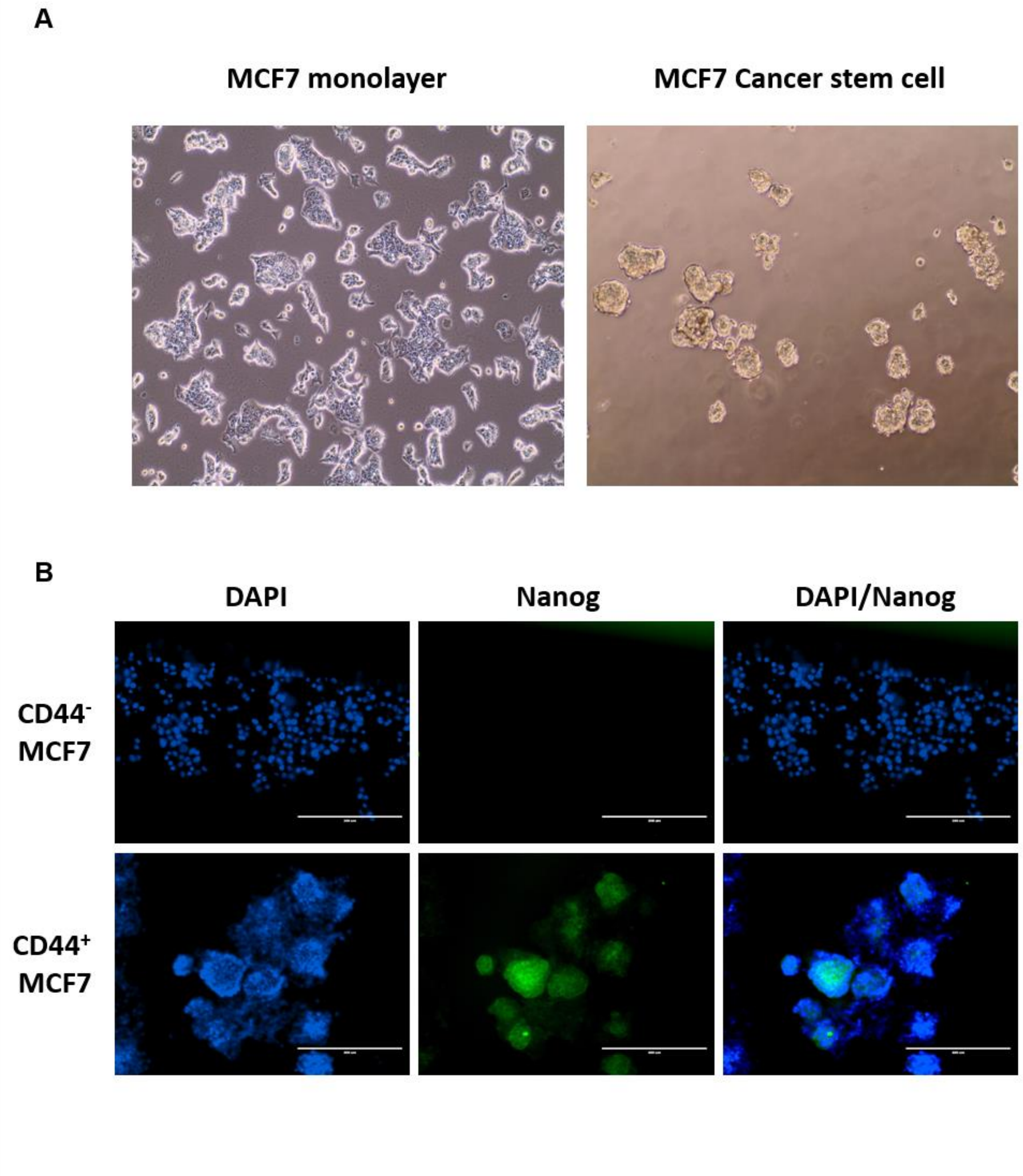

Supplementary Figure 1. Characterization of isolated CD44⁺ CSCs. (A) MCF7 monolayer cells and isolated MCF7 CSCs using CD44-positive magnetic beads are shown. (B) Confocal microscopic images revealed the expression of Nanog as a stemness marker. The bars indicate 400 µm. The nucleus was visualized by DAPI staining.
